# Intermittent Induction of HIF-1α Produces Lasting Effects on Malignant Progression Independent of Its Continued Expression

**DOI:** 10.1371/journal.pone.0125125

**Published:** 2015-04-20

**Authors:** Hyunsung Choi, David L. Gillespie, Shauna Berg, Christopher Rice, Sandrine Couldwell, Jie Gu, Howard Colman, Randy L. Jensen, L. Eric Huang

**Affiliations:** 1 Department of Neurosurgery, Clinical Neurosciences Center, University of Utah, Salt Lake City, Utah, United States of America; 2 Department of Oncological Sciences, Huntsman Cancer Institute, University of Utah, Salt Lake City, Utah, United States of America; University of Pécs Medical School, HUNGARY

## Abstract

Dysregulation of hypoxia-inducible transcription factors HIF-1α and HIF-2α correlates with poor prognosis in human cancers; yet, divergent and sometimes opposing activities of these factors in cancer biology have been observed. Adding to this complexity is that HIF-1α apparently possesses tumor-suppressing activities, as indicated by the loss-of-function mutations or even homozygous deletion of *HIF1A* in certain human cancers. As a step towards understanding this complexity, we employed 8-week intermittent induction of a stable HIF-1α variant, HIF1α(PP), in various cancer cell lines and examined the effects on malignant progression in xenografts of immunocompromised mice in comparison to those of HIF2α(PP). Although 8-week treatment led to eventual loss of HIF1α(PP) expression, treated osteosarcoma U-2 OS cells acquired tumorigenicity in the subcutaneous tissue. Furthermore, the prior treatment resulted in widespread invasion of malignant glioma U-87 MG cells in the mouse brain and sustained growth of U-118 MG glioma cells. The lasting effects of HIF-1α on malignant progression are specific because neither HIF2α(PP) nor β-galactosidase yielded similar effects. By contrast, transient expression of HIF1α(PP) in U-87 MG cells or constitutive expression of HIF1α(PP) but not HIF2α(PP) in a patient-derived glioma sphere culture inhibited tumor growth and spread. Our results indicate that intermittent induction of HIF-1α produces lasting effects on malignant progression even at its own expense.

## Introduction

Malignant tumors encounter conditions of low oxygen and nutrient deprivation as they progress. These adverse conditions, albeit detrimental to tumor growth, are associated with tumor progression and resistance to chemo- and radiotherapies. Since its initial discovery as a nuclear factor that binds to the human erythropoietin gene [1], the hypoxia-inducible transcription factor HIF-1 has been recognized as a major regulator that enables cells to overcome the severe microenvironmental stress in tumor development [2–9].

HIF-1 is a heterodimer consisting of HIF-1α and ARNT (aryl hydrocarbon receptor nuclear translocator) [10], and its activation depends primarily on the oxygen-sensitive HIF-1α subunit [11,12], which is degraded through the ubiquitin—proteasome pathway upon recognition by the von Hippel-Lindau (VHL) protein as part of the E3 ubiquitin ligase [13–17]. The VHL protein binds to HIF-1α and its paralog HIF-2α by recognizing two highly conserved, hydroxylated proline residues (HIF-1α Pro-402 and Pro-564, and HIF-2α Pro-405 and Pro-531) for polyubitylation [18–20]. Hypoxia inhibits prolyl hydroxylation, thereby preventing HIF-1α degradation. Subsequently, stabilized HIF-1α and HIF-2α undergo nuclear translocation, dimerization with ARNT, and recruitment of the transcription coactivators p300/CBP, resulting in transcriptional activation of a series of genes for angiogenesis, metabolism, and survival.

Whereas HIF-1α is ubiquitously expressed, HIF-2α expression seems restricted to certain tissues in development and physiology [21,22]. The abundance of HIF-1α as well as HIF-2α is frequently detected in the vast majority of human cancers [2–7,23]. Although these transcription factors were initially thought to share overlapping functions in tumor progression, each seems to possess unique and sometimes opposing activities through specific target gene activation and differential interactions with other proteins [24–26]. Specifically, their opposing activities have been shown in the regulation of cell cycle and DNA repair: Whereas HIF-1α inhibits cell-cycle progression and DNA repair by antagonizing c-Myc activities, HIF-2α does the reverse by enhancing c-Myc activities [26–28].

Furthermore, the roles of HIF-1α and HIF-2α in cancer seem context dependent. Whereas HIF-2α acts as a tumor suppressor in glioma, non-small cell lung cancer, and hepatocellular carcinoma [29–31], it drives tumorigenesis and growth of VHL-deficient renal clear-cell carcinoma [32]. In keeping with this, *EPAS1* (encoding HIF-2α) polymorphisms have been identified as one of the two susceptibility loci in renal cell carcinoma [33]. In addition, somatic, gain-of-function mutations in HIF-2α have been linked to the development of paraganglioma and somatostatinoma in patients [34]. Likewise, HIF-1α has been implicated as a tumor suppressor especially in kidney cancer [35], even though substantial evidence in the literature support a critical role of HIF-1α in progression and metastasis [7]. The tumor-suppressing activity of HIF-1α is strongly indicated by the genetic evidence that focal, homozygous deletions of *HIF1A* gene are found in many VHL-deficient renal clear-cell carcinoma cell lines and the functional evidence that HIF-1α inhibits cell proliferation and tumor growth [35]. All these studies suggest complex roles for HIF-1α and HIF-2α in cancer.

As a step towards understanding the complexity of cancer biology, we employed intermittent induction of HIF-1α and HIF-2α in various cancer cell types and investigated their differential effects on malignant progression in immunodeficient mice.

## Materials and Methods

### Plasmid construction and viral production

An oxygen-resistant HIF-1α, HIF1α(PP), with P402A and P564A substitutions [36], was cloned into pLenti6.3/TO/V5-DEST through homologous recombination reactions (Invitrogen, Carlsbad, CA, USA). Similarly, HIF-2α(PP) with P405A and P531A substitutions and a 3xFLAG at the amino terminus was cloned into the same vector. To produce lentiviruses, 293FT cells (Invitrogen) derived from a human embryonic kidney cell line were transfected with a lentiviral vector and Virapower packaging mix (Invitrogen) using Lipofectamine 2000. Lentiviral supernatant was harvested 3 days after transfection and filtered through a 0.45-μm sterile syringe filter (VWR, Radnor, PA, USA). The filtered virus was aliquoted and stored at −80°C. Viral titers were determined according to the manufacturer’s instruction.

### Lentiviral transduction

To establish tetracycline-regulated stable cell lines, we used the ViraPower HiPerform T-REx Gateway Vector kit (Invitrogen) following the manufacturer’s instruction. Tetracycline regulation in the T-REx system is based on the binding of tetracycline to the tetracycline repressor and derepression of the promoter controlling expression of either HIF1α(PP) or HIF2α(PP). Cells were infected at 5 multiplicity of infections (MOIs) with the lentivirus expressing tetracycline repressor and selected with geneticin at 500 μg/ml for U-2 OS, 200 μg/ml for U-87 MG, and 400 μg/ml for U-118 MG. These cells were then infected at 2 MOIs with a lentivirus derived from any of the pLenti6.3/TO/V5-DEST constructs and selected with blasticidin at 5 μg/ml for U-2 OS and U-118 MG, and 2 μg/ml for U-87 MG. Selected cells were pooled and used for further studies.

### Cell culture and intermittent induction with tetracycline

U-2 OS, U-87 MG, and U118MG were purchased from the American Type Culture Collection (Rockville, MD, USA). U-2 OS and U-118 MG cells were maintained in Dulbecco’s modified Eagle’s medium supplemented with 10% fetal bovine serum and penicillin/streptomycin. U-87 MG cells were maintained in minimum essential medium with supplements as above. Cell culture conditions were maintained at 37°C and 5% CO_2_. For intermittent induction of the gene of interest, tetracycline was administered weekly at 1 μg/ml on day 1 and removed on day 4 for a total of 8 weeks.

### Western blot

Cell extract was prepared in a lysis buffer [11] which contains 20 mM HEPES, pH 7.9, 0.42 M NaCl, 1.5 mM MgCl_2_, 0.2 mM EDTA, 25% glycerol freshly supplemented with 0.5 mM DTT and protease inhibitor cocktail (Roche, Mannheim, Germany). Protein concentrations were determined by using Pierce BCA protein assay kit (Pierce, Rockford, IL, USA). Antibodies used for Western blotting were mouse anti-human HIF-1α (#610959, BD Bioscience, San Jose, CA, USA), rabbit anti-V5 antibody, and anti-β-tubulin (#V8137 and #T0198, Sigma-Aldrich, St. Louis, MO, USA). Signals were developed using Super Signal West Pico chemiluminescent substrate (Cat#34018, Thermo Scientific, Rockford, IL, USA).

### Gene expression, cell proliferation, and anchorage-independent growth

For reporter assays, 293T cells were seeded in 24-well plates and transiently cotransfected with 400 ng pEpoE-luc [11] and 200 ng pLenti-HIF1α(PP) or pLenti-HIF2α(PP), as well as 50 ng pCMV-EGFP for normalization. pLenti-LacZ was used as a control. Twenty-four hours after transfection, cells were lysed and assayed for reporter activity in the Bright-Glo Luciferase assay system (Promega, Madison, WI, USA) according to the manufacturer’s instructions. Reagents for TaqMan gene expression were purchased from Invitrogen and real-time PCR reactions were performed according to the manufacturer’s instructions. Assays for cell proliferation and anchorage-independent growth were described previously [37].

### Tumor transplantation

All animal studies were performed according to the protocol (13–09003) approved by the University of Utah Institutional Animal Care and Use Committee. There were six mice in each group or otherwise indicated. For subcutaneous injections, treated U-2 OS (5×10^6^) cells were suspended in 100 μl of phosphate-balanced saline (PBS) per injection and grafted into the flanks of 6–8-week-old non-obese diabetic/severe-combined immunodeficient IL-2Rg-null (NOD/SCID gamma (NSG)) male mice. Mice were sacrificed and tumor was extracted when the diameter reached 1 cm. For intracranial implantation, treated U-87 MG or U-118 MG (7×10^5^) cells in a total volume of 5 μl were mixed with BD Matrigel basement membrane matrix (BD Biosciences). NSG mice were anesthetized with isoflurane, and injections were positioned at 2 mm right to the bregma and 1 mm anterior to the coronal suture, with 2 mm depth. Approximately 5–7 weeks after injection, mice were sacrificed and the brain was extracted for histological examination.

### Bioluminescent imaging

Mice were injected intraperitoneally with a 50 mg/kg D-luciferin and 100 mg/kg ketamine/10 mg/kg xylazine (Sigma) mixture. Images were acquired 10 min after injection with the IVIS 100 or 200 imaging system (Xenogen, Alameda, CA, USA). Quantitative analysis of bioluminescent intensity from the images was performed using LivingImage software (Xenogen).

### Glioma Stem Cell Culture, Transduction, and Intracranial Implantation

U-87 MG, U-118 MG, and patient-derived glioma sphere culture cells, GSC20 [38], were maintained in DMEM/F12 medium supplemented with B27, 20 ng/ml of EGF and 20 ng/ml of bFGF (Invitrogen). To maintain spheric growth, fresh medium with bFGF and EGF at ~10–20% of the total volume was added twice per week. Spheres were triturated with Accutase (Invitrogen, Cat: A11105-01) and fed with fresh culture medium with supplement. Lentiviral transduction was performed after trituration at 1 MOI. Intracranial implantation of GSC20 was performed with 2×10^5^ cells per injection.

### Statistical analysis

Statistical differences between groups of data were determined in a t-test of two tails. n = 3 or greater as indicated was used for each data set. Statistical significance was indicated as *, p-value < 0.05; **, p-value < 0.01; and ***, p-value < 0.001.

## Results

### Regulated HIF-α expression in various cancer cell lines

**Fig 1:**
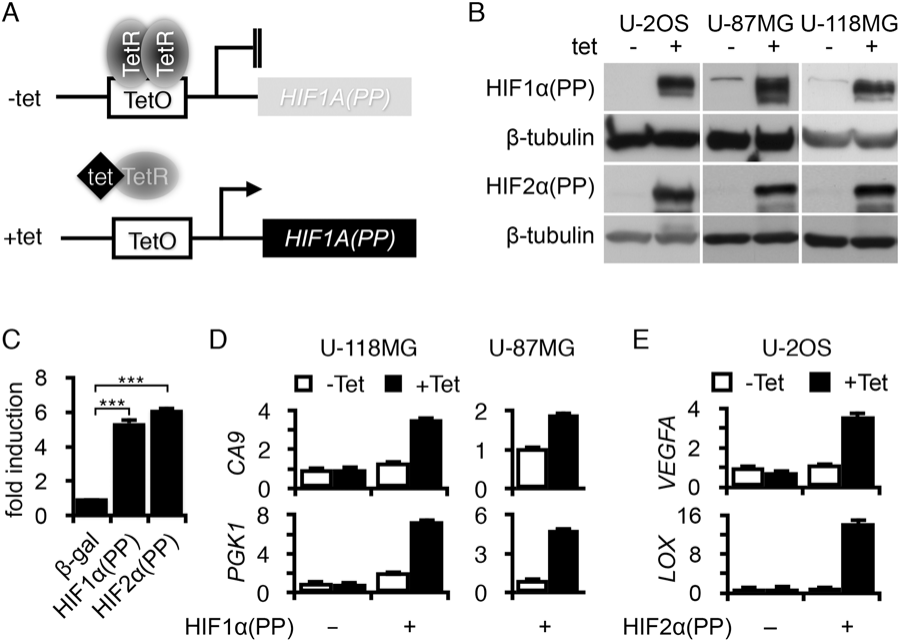
Tetracycline regulation of HIF1α(PP) and HIF2α(PP) expression and transcriptional activity. (A) Tetracycline regulation is diagrammed where the addition of tetracycline (tet) results in dissociation of tetracycline repressor (TetR) from the tetracycline operon (TetO) and, in turn, gene activation. (B) Western blot analysis of transduced cell types, as indicated, for the expression of HIF1α(PP) and HIF2α(PP) after 2-day treatment with tetracycline. (C) Transcriptional activities of HIF1α(PP) and HIF2α(PP) were tested in a reporter assay in reference to β-galactosidase (β-gal). ***, *p*-value < 0.001. (D, E) The expression of HIF target genes (*PGK1*, *CA9*, *VEGFA*, and *LOX*) was analyzed in specified cell lines by using real-time PCR after 2-day treatment with tetracycline.

To better investigate the role of HIF-α in cancer, we developed a tetracycline-regulated gene expression system through lentiviral transduction (Fig 1A) of the cancer cell lines used below. Prolyl hydroxylation sites (Pro-402 and Pro-564) in HIF-1α [18–20] were replaced with alanine to generate a stable HIF-1α variant, HIF1α(PP). The HIF-2α equivalent, HIF2α(PP), was generated similarly. Human cancer cell lines including U-2 OS of osteosarcoma and U-87 MG and U-118 MG of glioblastomas were used for HIF-α expression. As a control, a lentivirus expressing β-galactosidase (β-gal) was included. Cells were pooled after selection and analyzed for gene expression.

As expected, the addition of tetracycline resulted in robust induction of HIF1α(PP), HIF2α(PP), and β-gal in all the cell lines examined by Western blot analysis (Figs 1B and S1). Furthermore, both HIF1α(PP) and HIF2α(PP) were transcriptionally active in stimulating an erythropoietin reporter gene, *EPO-luc*, by five- and sixfold, respectively (Fig 1C). Moreover, induction of HIF1α(PP) upregulated target genes *CA9* (carbonic anhydrase IX) [39] and *PGK1* (phosphoglycerate kinase 1) [40] (3- and 6-fold in U-118 MG and 2- and 5-fold in U-87 MG) (Fig 1D). Similarly, HIF2α(PP) also increased expression of *VEGFA* (vascular endothelial growth factor A) [41] and *LOX* (lysyl oxidase) [42] by 4- and 16-fold, respectively, in U-2 OS (Fig 1E).

### Intermittent induction of HIF-1α transgene leads to eventual loss of expression

Although hypoxia has long been known to enhance cancer metastatic potential [43,44], intermittent hypoxia, defined as repeated cycles of hypoxia and reoxygenation, seems more effective than prolonged hypoxia in enhancing spontaneous metastasis [45]. Recent studies indicate intermittent hypoxia is a key regulator of the interplay between cancer cell and endothelial cell for tumor angiogenesis and growth and resistance to chemo- and radiotherapy [46]. Furthermore, HIF-1α levels seem well maintained during the cycling [47].

**Fig 2:**
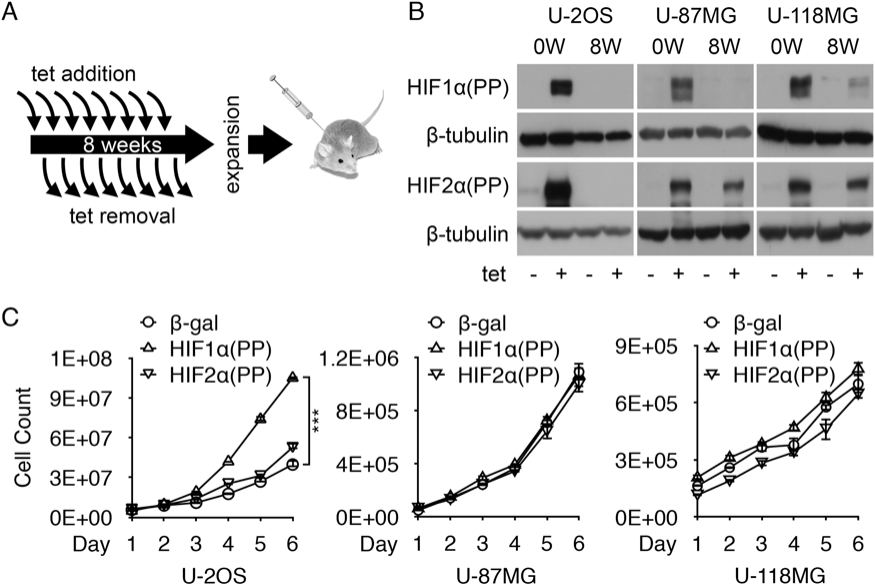
Intermittent induction resulted in loss of of HIF1α(PP) expression. (A) Intermittent induction involves the administration of tetracycline into cell culture each week on day 1 and removal on day 4 each week for a total of 8 weeks. Afterwards, cells were allowed to expand for further analyses and injections. (B) After intermittent induction (8W), different types of cells as indicated were induced again with tetracycline for 2 days and analyzed by Western blotting in reference to those without intermittent induction (0W). (C) Cell proliferation was determined by cell counting after intermittent induction. ***, *p*-value < 0.001.

To reproduce intermittent hypoxia in cancer, we elected to treat the transduced cells with tetracycline for 3 days and without for 4 days every week for 8 weeks (Fig 2A). Interestingly, at the end of the treatment most of these treated cells no longer or barely responded to tetracycline (Fig 2B). Specifically, HIF1α(PP) expression was essentially lost at protein levels in all cell lines, while various degrees of HIF2α(PP) attenuation were observed. However, β-gal expression remained inducible (S1 Fig). The loss at protein levels correlated with that at transcript levels. To exclude the possibility of slow recovery, we continued to culture these cells for additional weeks in the absence of tetracycline and found no recovery of HIF1α(PP) expression. Although the underlying mechanism requires further investigation, we reasoned that the loss of HIF1α(PP) induction would help us determine the consequence of intermittent induction, rather than *de novo* expression, of HIF1α(PP) on malignant progression.

### U-2 OS cells acquire tumorigenicity in the absence of continued expression of HIF-1α variant

**Fig 3:**
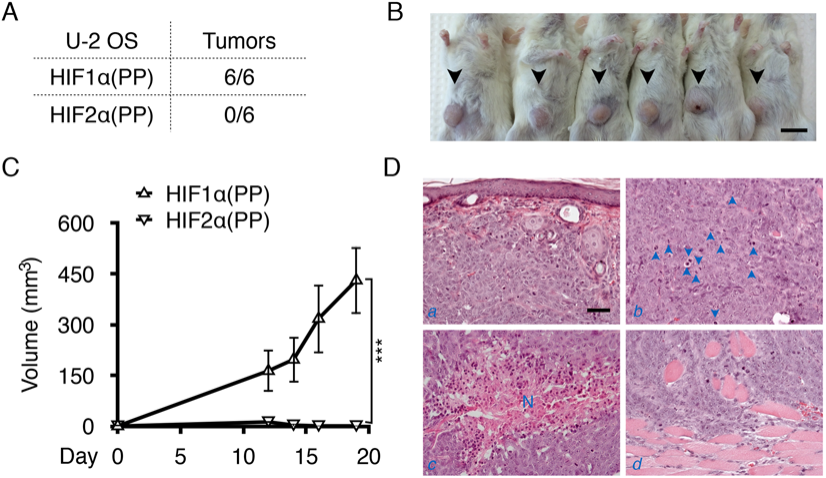
U-2 OS cells acquired tumorigenicity after intermittent induction of HIF1α(PP). (A) Tumor incidence is shown in 6 NSG mice after bilateral, subcutaneous injections of the 8-week HIF1α(PP) and HIF2α(PP) cells. (B) Only injections of the HIF1α(PP) cells produced tumors, as indicated by arrowheads. Scale bar, 1 cm. (C) Tumor volume was calculated based on measurements and plotted as a function of time. ***, *p*-value < 0.001. (D) Hematoxylin and eosin staining of the tumor specimens reveals invasion of the dermal layer (*a*), numerous mitoses (arrowheads) (*b*), necrosis (N) (*c*), and invasion into the striated muscle layer (*d*). Scale bar, 100 μm.

We first observed that the HIF1α(PP)-induced U-2 OS cells grew twofold faster than the β-gal control without further induction, whereas only a modest increase was detected in the HIF2α(PP)-induced cells (Fig 2C). Importantly, the former but not the latter became tumorigenic when injected subcutaneously into the flanks of NSG mice; all 6 injections with the HIF1α(PP) cells resulted in tumor formation, whereas none of the HIF2α(PP) cells did at the contralateral sites (Fig 3A and 3B). Of note, none of the β-gal controls became tumorigenic. Moreover, the HIF1α(PP) tumors grew at an exponential pace (Fig 3C). Histological examination confirmed the malignant growth of these tumors, as indicated by hypercellularity and necrosis, increased mitosis, and invasion into the dermis and the striated muscle layers (Fig 3D). Similar data were obtained when CD1 nude mice were used. Thus, we conclude that intermittent induction of HIF1α(PP), but not HIF2α(PP), programs the non-tumorigenic U-2 OS cells for malignant progression independent of its continued expression.

### HIF-1α promotes invasion of glioma cells in the mouse brain

To extend this original observation, we focused on glioma progression and employed an orthotopic tumor model through intracranial injections. Unlike their U-2 OS counterpart, the U-87 MG and U-118 MG cells with prior HIF1α(PP) induction exhibited a similar growth rate in culture as their controls (Fig 2C), suggesting variation of HIF1α(PP) effects after intermittent induction. Furthermore, HIF1α(PP)-induced U-87 MG cells showed ~10-fold increase in the development of tumor spheres but no increase in anchorage-independent growth (S2 Fig). Yet, a decrease in tumor sphere formation and anchorage-independent growth was observed in HIF1α(PP)-induced U-118 MG cells.

**Fig 4:**
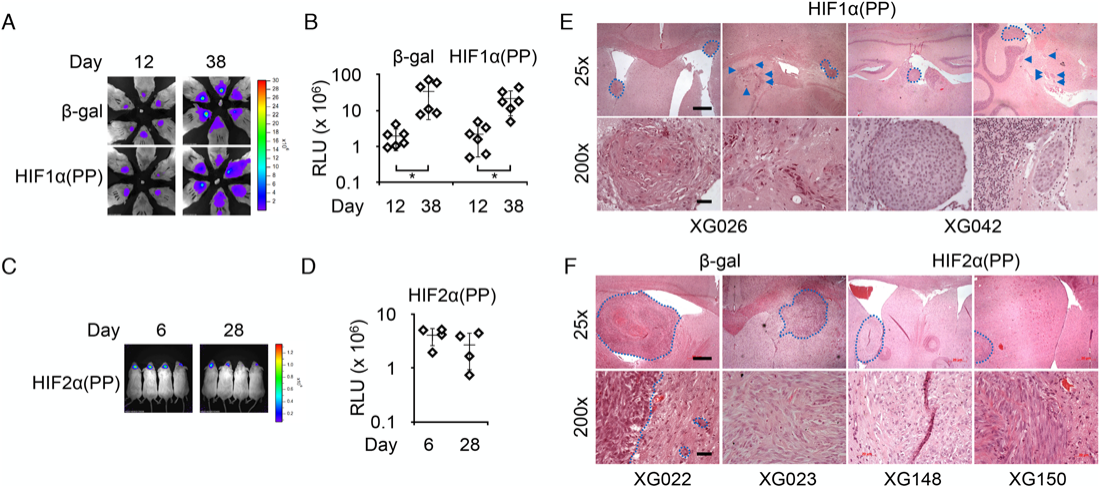
Intermittent induction of HIF1α(PP) promoted intracranial spread of U-87 MG cells. Bioluminescent imaging analysis of β-gal and HIF1α(PP)-derived intracranial tumors (A) and HIF2α(PP)-derived intracranial tumors (C). The respective tumor volumes were calculated based on the relative luminescent units (RLU) and plotted in a log scale (B and D). *, *p*-value < 0.05. (E) HIF1α(PP)-derived tumors had small yet numerous lesions invading the Ammon’s horn of the hippocampal region (XG026) and hindbrain and cerebellum (XG042), as indicated by arrowheads. (F) β-gal- and HIF2α(PP)-derived tumors were large and often singular in the cerebral cortex. Tumor lesions are demarcated in dash lines. Hematoxylin and eosin—stained images are presented at 25× and 200× magnifications, with scale bars of 1 mm and 100 μm, respectively.

Original U-87 MG cells are tumorigenic after intracranial transplantation. Interestingly, during a ~5-week monitoring of tumor growth using bioluminescent imaging, we found that the increase in tumor volume from the β-gal-induced cells was equivalent to, if not greater than, that from the HIF1α(PP)-induced cells (Fig 4A and 4B). By contrast, tumors derived from the HIF2α(PP) cells grew poorly and failed to expand during the experimental period (Fig 4C and 4D). The lack of tumor growth from the HIF2α(PP) cells appears consistent with a previous report that HIF-2α acts as a tumor suppressor in glioma [29].

Although prior intermittent induction of HIF1α(PP) had hardly any effect on U-87 MG cell proliferation in culture and *in vivo*, it is noticeable that the bioluminescent signals from the derived tumors permeated broadly beyond the frontal lobes of the brain (Fig 4A), indicative of spreading of tumor cells. Indeed, tumor lesions from the HIF1α(PP) cells were numerous foci throughout the brain (Figs 4E and S3B); in addition to the cerebral cortex, invasions were identified in the hypothalamus, midbrain, hindbrain, and even cerebellum. By contrast, tumor lesions derived from the β-gal control tended to be singular, large, and localized to the cerebral cortex (Fig 4F), even though very few developed more than single lesions with distal invasions (S3A Fig). Furthermore, only small, singular lesions were found in all HIF2α(PP)-derived tumors (Fig 4F). Histological examination at high magnification revealed aggressive invasion of round cells in the HIF1α(PP)-derived tumors, whereas relatively low-density, spindle-shaped tumor cells were frequently observed interwoven with fibrous tissues in β-gal and HIF2α(PP) tumors (Fig 4E and 4F). However, all three cell types appeared indistinguishable in culture.

**Fig 5:**
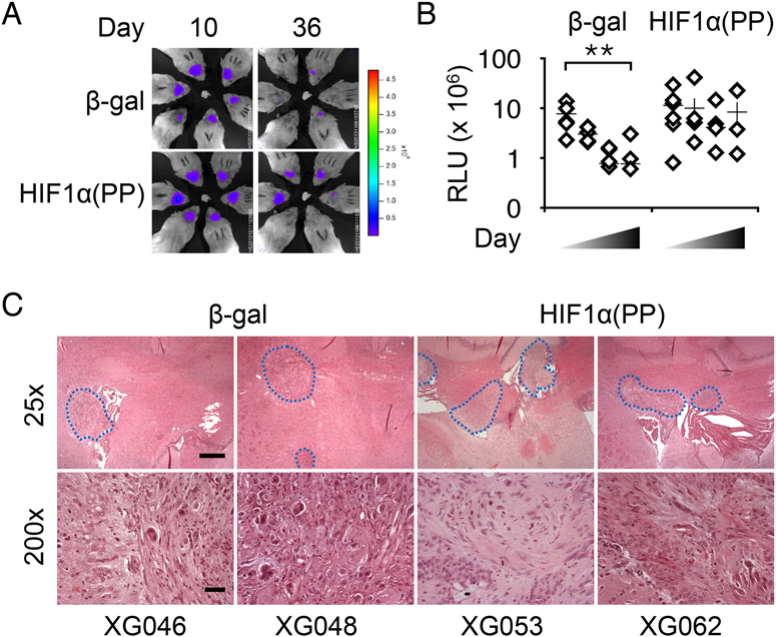
Intermittent induction of HIF1α(PP) facilitated intracranial tumor growth of U-118 MG cells. (A) Bioluminescent imaging analysis of intracranial tumors derived from 8-week treated cells as indicated. (B) Tumor volumes, plotted in a log scale, from β-gal cells decreased significantly during the course of 49 days, but those from HIF1α(PP) did not. **, *p*-value < 0.01. (C) Representative tumor lesions are shown in hematoxylin and eosin staining from 2 individual mice of each group at 25× and 200× magnifications, with scale bars of 1 mm and 100 μm, respectively.

With respect to U-118 MG, 8-week intermittent induction of HIF1α(PP) failed to result in discernible morphological differences in culture but apparently helped tumor maintenance (Fig 5A and 5B), as indicated by the preserved bioluminescent signals throughout a 7-week period of observation. In contrast, the signals from the β-gal control were diminished progressively, resulting in a statistically significant drop in tumor volume (Fig 5B). Tumor incidence of the HIF1α(PP) cells was 5/6, similar to that of the control (4/6). Furthermore, tumors from the HIF1α(PP) cells often had multiple lesions, in contrast to mostly single lesions in the β-gal control group. Therefore, these results further corroborate the long-lasting effects of HIF-1α on malignant progression and the differential observations between cell culture and animal models.

### Transient induction of HIF-1α inhibits glioma growth

**Fig 6:**
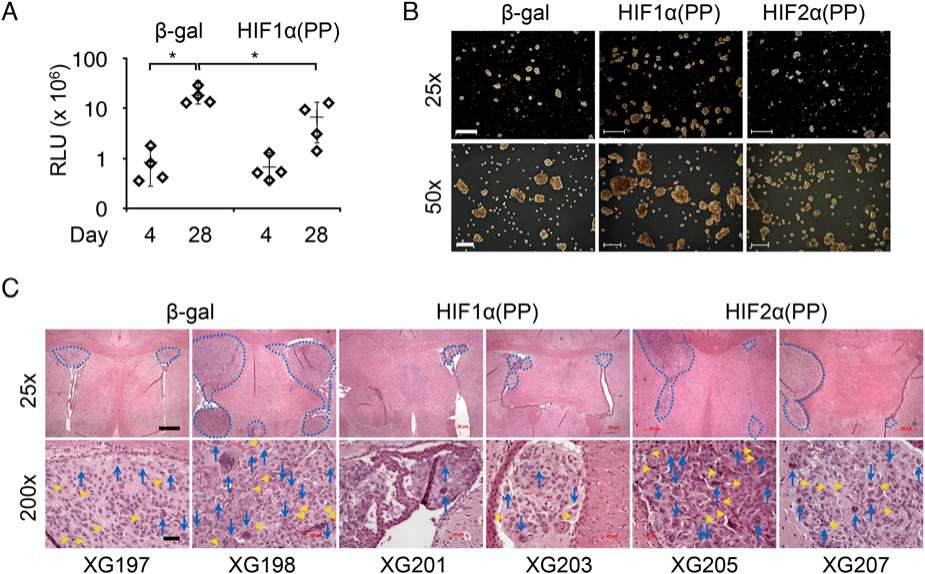
Transient expression of HIF1α(PP) inhibited intracranial tumor growth. (A) U-87 MG cells were induced for 2 days prior to intracranial injections. Tumor volumes, plotted in a log scale, decreased significantly from HIF1α(PP) in reference to β-gal. (B) After infection with lentiviruses expressing gene products as indicated, GSC20 cells were grown in neurobasal-A medium and images of tumor spheres were captured at 25× and 50× magnifications, with scale bars of 1 mm and 400 μm, respectively. (C) Representative tumor lesions from 2 individual mice of each group are presented at 25× and 200× magnifications, with scale bars of 1 mm and 100 μm, respectively. Tumor lesions are demarcated in dash lines. Mitoses are indicated by yellow arrowheads and multi-nucleation by blue arrows.

To test whether transient induction of HIF1α(PP) would produce similar effects *in vivo*, we analyzed tumor growth of U-87 MG cells that had been treated with tetracycline in culture for only 2 days before intracranial transplantation. Fig 6A shows that transient induction of HIF1α(PP) retarded tumor growth significantly in the brain in reference to that of β-gal. To extend this finding, we employed a patient-derived, mesenchymal glioma sphere culture, GSC20 [38]. These cells were allowed for constitutive expression of β-gal, HIF1α(PP), or HIF2α(PP) after lentiviral infection and grown in sphere culture medium for adequate expansion prior to intracranial injections. It is noteworthy that while the HIF1α(PP) population expanded slightly faster than the β-gal control and more readily formed tumor spheres, the HIF2α(PP) multiplied at an extremely slow rate (Fig 6B).

Contrary to these *in vitro* findings, injection of the HIF1α(PP) cells led to the development of only small, primarily singular tumors in the mouse brain, whereas the other two cell types yielded much larger, multiple lesions and invasions (Figs 6C and S4). Tumor incidence was slightly decreased for HIF1α(PP) cells (3/5 vs 4/5 for the other two cell types). Strikingly, tumors derived from the β-gal and HIF2α(PP) cells manifested a multitude of mitoses and multi-nuclear giant cells, both of which were markedly diminished in HIF1α(PP)-derived tumors (Figs 6C and S4). Furthermore, vascular proliferation and necrosis, common features of glioblastomas [48], were present in tumors derived from the β-gal and HIF2α(PP) cells but not the HIF1α(PP) cells (S4 Fig). Taken together, these results suggest that HIF-1α can also inhibit glioma growth and progression.

## Discussion

We provide evidence in this study that intermittent induction of HIF1α(PP) *in vitro* produced lasting effects on malignant progression of different cancer cell lines, unexpectedly independent of continued expression of the transgene. All these cells eventually lost HIF1α(PP) expression after continuous culture, yet retained the acquired malignant traits in the *in vivo* setting. These results indicate that repeated activation of HIF-1α can program cancer cells to acquire perpetual signaling possibly through feed-forward biochemical/metabolic loops or genetic/epigenetic changes, even though the underlying mechanism requires further investigation. The study also suggests that HIF-1α can promote malignant progression at its own expense, which might account for HIF-1α inactivation in human cancer.

Although repeated cycles of hypoxia and reoxygenation have long been known to promote tumor progression [45], our experimental system allows us to directly interrogate HIF-1α and HIF-2α, excluding other possible aspects such as changes in reactive oxygen species, which may also contribute to malignant progression. It should be noted that the cancer cells treated for 8 weeks in our study, although they no longer expressed HIF1α(PP), did maintain endogenous HIF-1α expression under hypoxia, a potential contributing factor to the process and a disadvantage of our experimental system. Interestingly, knockdown of endogenous HIF-1α by short-hairpin RNA in the cancer cells treated for 8 weeks impeded tumor growth but failed to prevent invasion, suggesting that intermittent induction of HIF1α(PP) in culture is key to programming glioma cells for invasion whereas endogenous HIF-1α potentially facilitates tumor growth. This interpretation is consistent with our results that tumors from the control groups, β-gal and HIF2α(PP), exhibited much less invasion despite endogenous HIF-1α expression; however, we cannot exclude the possibility that endogenous HIF-1α was required for programming cancer cells during intermittent induction of HIF1α(PP). This possibility will be better addressed in HIF-1α-deficient cancer cells or conditional genetic models.

It is interesting to note that GSC20-derived tumor cells in the control group featured rampant mitoses and numerous giant-sized, hyperchromatic nuclei, indicative of rapid cell proliferation and aberrant DNA replication; however, HIF1α(PP) expression not only retarded tumor growth but also markedly reduced mitosis of tumor cells and diminished multi-nucleation. These effects are in agreement with the inhibitory roles of HIF-1α, but not HIF-2α, in cell-cycle progression and DNA replication upon transient induction [49–51]. Although these findings are reminiscent of previous reports that HIF-1α retards tumor growth [52,53], our data collectively indicate that HIF-1α has pleotropic effects that are context dependent.

Our study also indicates that HIF-1α is a potent inducer of glioma invasion. This is particularly interesting because investigations of glioma invasion have been hampered by the scarcity of representative experimental models [54]. Although we have not identified the signaling pathway leading to glioma invasion, it appears to be independent of the major signaling pathways such as PI(3)K-Akt and MAPK after intermittent induction. Glioma aggressiveness is associated with a mesenchymal phenotype that is regulated by the C/EBPβ and STAT3 transcription factors [55]. In addition, CHI3L1 (chitinase 3-like 1), also known as BRP-39/HCGP39/YKL-40 [56], is considered a reliable gene expression marker for the mesenchymal subclass and local invasiveness of glioblastomas [57–60]. However, we observed no increase in STAT3 phosphorylation in HIF1α(PP)-induced U-87 MG cells. Furthermore, we detected decreased CHI3L1 expression in the invasive lesions by immunohistochemistry. Therefore, further studies are warranted to understand the mechanism by which intermittent induction of HIF-1α drives malignant progression.

Targeting HIF-1 for cancer therapy was based originally on the critical roles of HIF-1α in cancer biology, the association of HIF-1α overexpression with increased patient mortality in various cancer types, and the marked effects on tumor growth by inhibiting HIF-1α activity in preclinical studies [3–5,61]. Numerous small-molecule inhibitors therefore have been identified in preclinical studies to inhibit HIF-1α via targeting various signaling pathways that regulate HIF-1α expression, degradation, dimerization, DNA binding, and transactivation [62]. Despite these advances, caution must be exercised for the use of these inhibitors as a potential therapeutic strategy owing to the complex role of HIF-1α and HIF-2α in cancer [63]. Therefore, an in-depth understanding of complex hypoxia biology in cancer will be key to precision targeting and therapeutic efficacy [64].

## Supporting Information

S1 Fig Loss of HIF1α(PP) expression after 8-week intermittent induction. U-87 MG variants, as specified, were induced with tetracycline for 2 days and analyzed by Western blotting with antibodies against V5 and β-tubulin. HIF1α(PP) cells post intermittent induction (8W) no longer responded to tetracycline induction in contrast to those prior to intermittent induction (0W).
(TIF)

S2 Fig Analysis of U-87 MG and U-118 MG cells treated for 8 weeks. HIF1α(PP)-induced cells were assayed for tumor sphere formation in reference to the control (Con) (A). Scale bar, 200 μm. Tumor spheres > 200 μm were quantified and plotted (B). Soft agar assays were performed for *in vitro* tumorigenicity (C). Scale bar, 200 μm. Colonies > 200 μm were quantified and plotted (D).
(TIF)

S3 Fig Intracranial tumor lesions derived from U-87 MG variants after intermittent induction. (A) Invasive lesions were seen occasionally in tumors derived from β-gal cells (XG021), presented at 25× and 200× magnifications, with scale bars of 1 mm and 100 μm, respectively. (B) Additional lesions of widespread invasion derived from HIF1α(PP) cells are shown (XG042). Scale bar, 100 μm. Tumor lesions are demarcated in dash lines.
(TIF)

S4 Fig Additional malignant features of intracranial tumors derived from transduced GSC20. Invasive lesions were identified in the hindbrain of NSG mice injected with β-gal-transduced GSC20 (XG198) and HIF2α(PP)-transduced GSD20 (XG205). Tumor lesions also contained necrosis (XG198) and invasion in the forth ventricle (XG205, arrowhead). Vascular proliferation and multi-nucleation were observed commonly in these tumor lesions. Images are presented at 25× and 200× magnifications, with scale bars of 1 mm and 100 μm, respectively.
(TIF)
