# Supplementary figures and images for: Intermittent Induction of HIF-1α Produces Lasting Effects on Malignant Progression Independent of Its Continued Expression
Source: PLoS One. 2015 Apr 20;10(4):e0125125. doi: 10.1371/journal.pone.0125125 (PMC4404255; doi:10.1371/journal.pone.0125125)

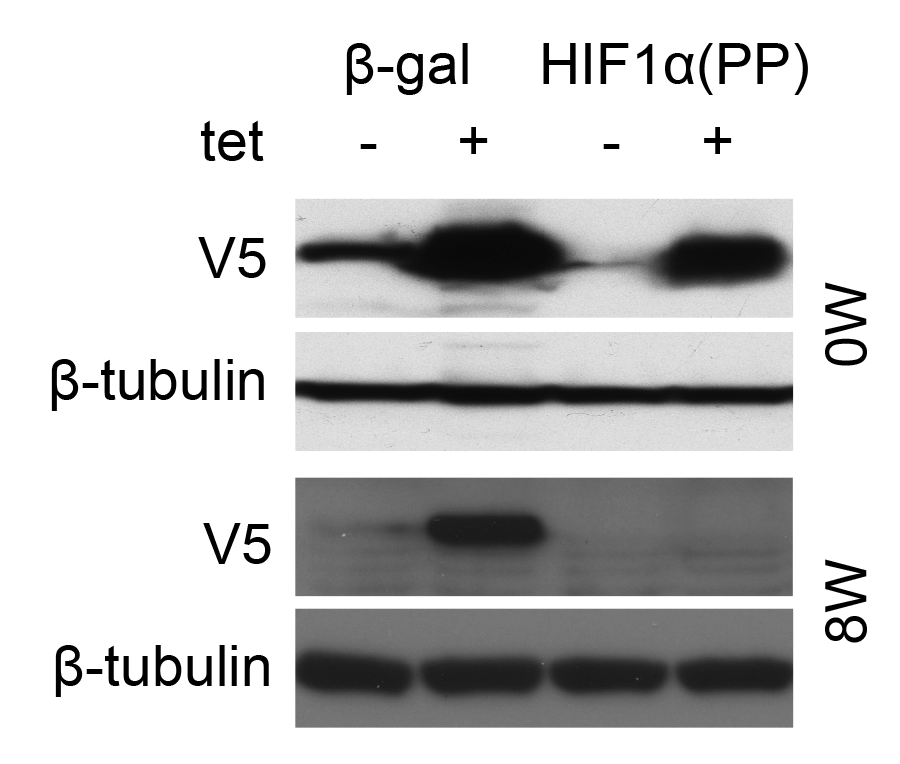

Supplement: S1 Fig — U-87 MG variants, as specified, were induced with tetracycline for 2 days and analyzed by Western blotting with antibodies against V5 and β-tubulin. HIF1α(PP) cells post intermittent induction (8W) no longer responded to tetracycline induction in contrast to those prior to intermittent induction (0W). (TIF) [file pone.0125125.s001.tif]

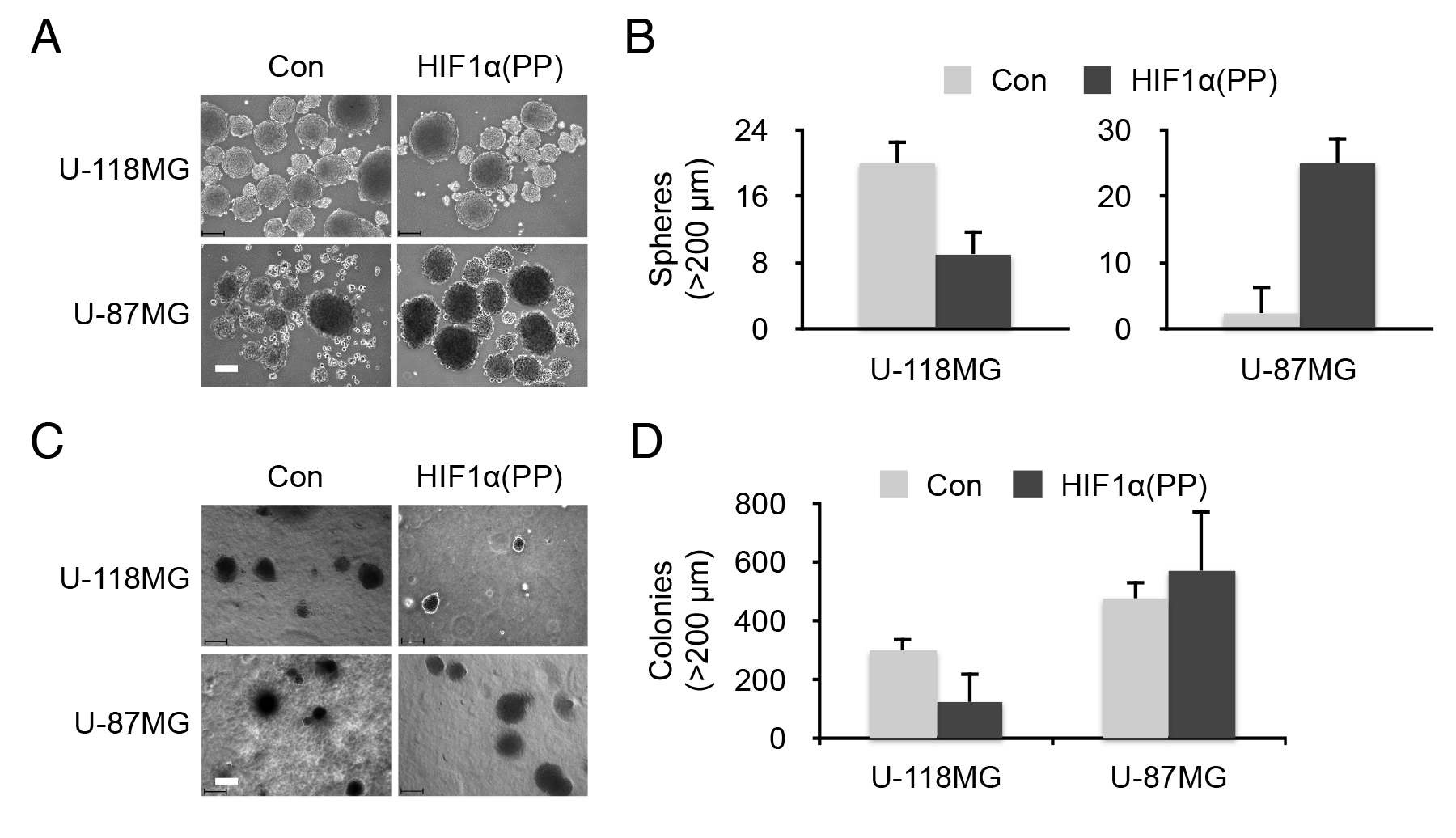

Supplement: S2 Fig — HIF1α(PP)-induced cells were assayed for tumor sphere formation in reference to the control (Con) (A). Scale bar, 200 μm. Tumor spheres > 200 μm were quantified and plotted (B). Soft agar assays were performed for in vitro tumorigenicity (C). Scale bar, 200 μm. Colonies > 200 μm were quantified and plotted (D). (TIF) [file pone.0125125.s002.tif]

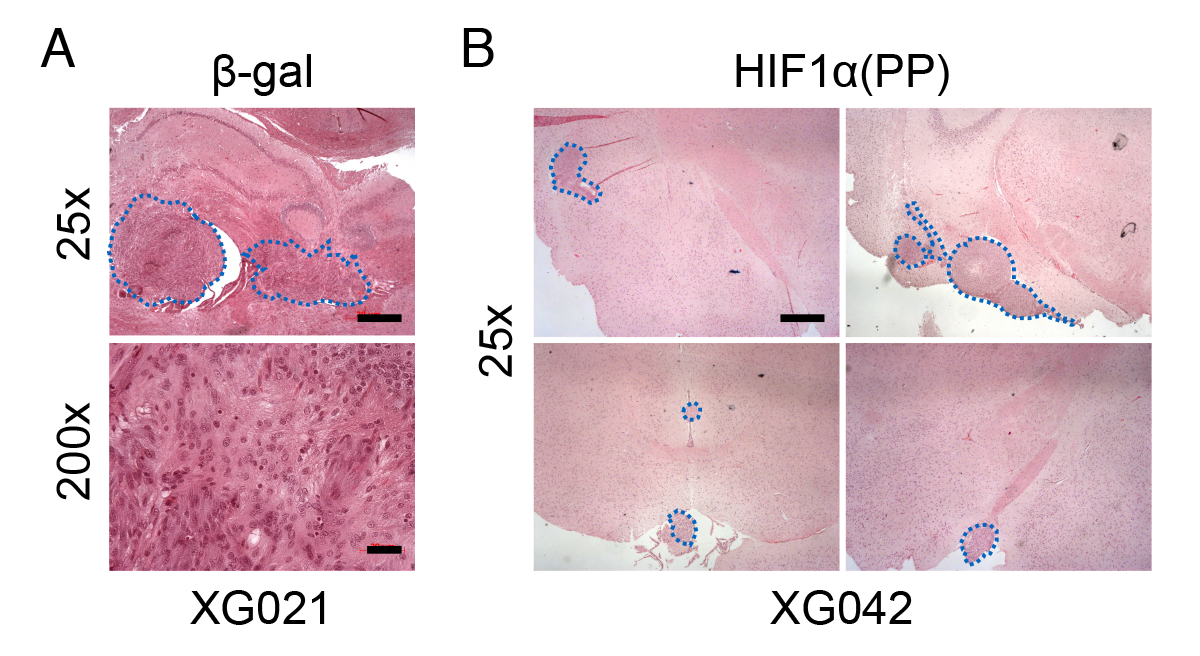

Supplement: S3 Fig — (A) Invasive lesions were seen occasionally in tumors derived from β-gal cells (XG021), presented at 25× and 200× magnifications, with scale bars of 1 mm and 100 μm, respectively. (B) Additional lesions of widespread invasion derived from HIF1α(PP) cells are shown (XG042). Scale bar, 100 μm. Tumor lesions are demarcated in dash lines. (TIF) [file pone.0125125.s003.tif]

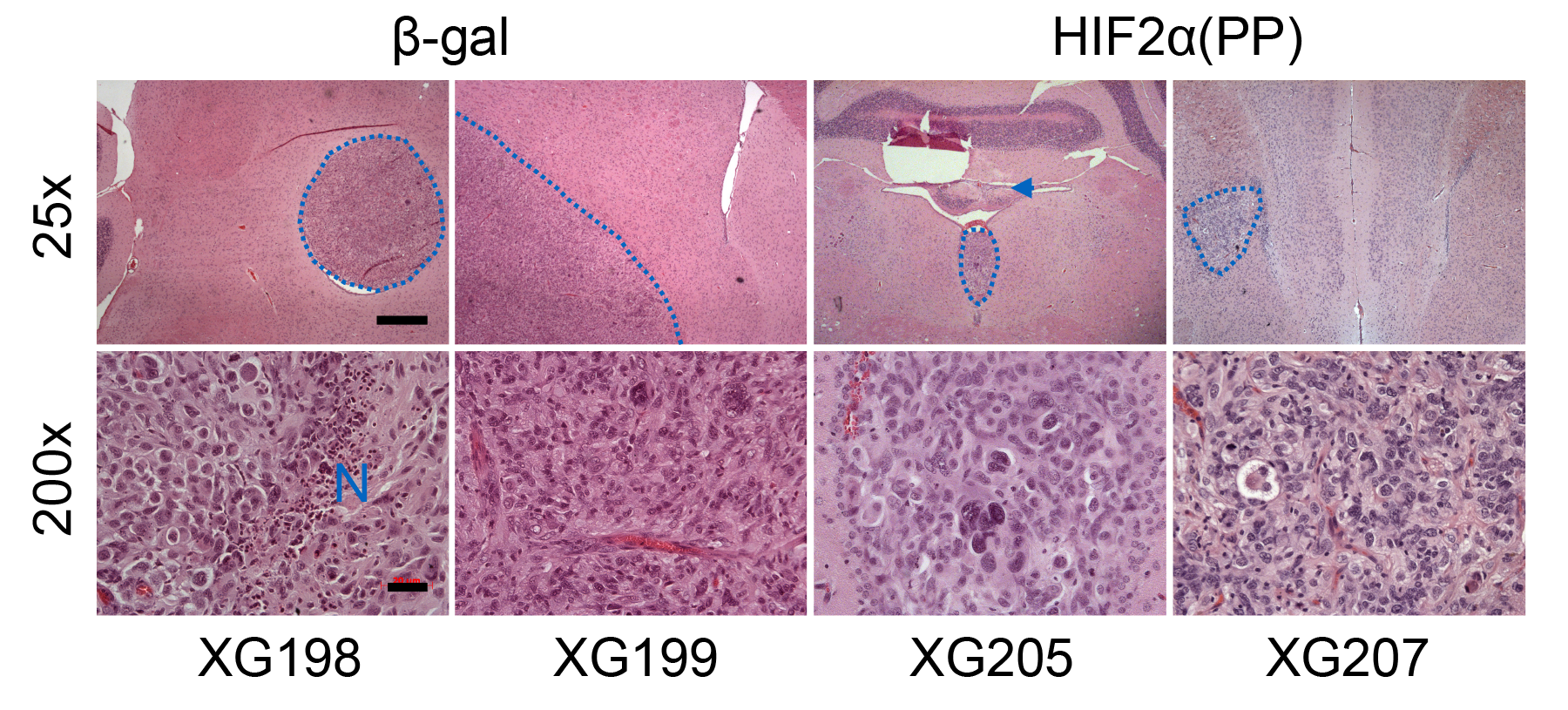

Supplement: S4 Fig — Invasive lesions were identified in the hindbrain of NSG mice injected with β-gal-transduced GSC20 (XG198) and HIF2α(PP)-transduced GSD20 (XG205). Tumor lesions also contained necrosis (XG198) and invasion in the forth ventricle (XG205, arrowhead). Vascular proliferation and multi-nucleation were observed commonly in these tumor lesions. Images are presented at 25× and 200× magnifications, with scale bars of 1 mm and 100 μm, respectively. (TIF) [file pone.0125125.s004.tif]
